# Anticoagulation Control in Older Atrial Fibrillation Patients Receiving Vitamin K Antagonist Therapy for Stroke Prevention

**DOI:** 10.1155/2022/5951262

**Published:** 2022-01-31

**Authors:** Hanis Zulkifly, Gregory Y. H. Lip, Deirdre A. Lane

**Affiliations:** ^1^University of Birmingham Institute of Cardiovascular Sciences, Sandwell and West Birmingham Hospitals NHS Trust, Birmingham, UK; ^2^Department of Pharmacy Practice, Fakulti Farmasi, Universiti Teknologi MARA (UiTM), Bandar Puncak Alam, Malaysia; ^3^Liverpool Centre for Cardiovascular Science, University of Liverpool, Liverpool, UK; ^4^Department of Clinical Medicine, Aalborg University, Aalborg, Denmark

## Abstract

**Introduction:**

Efficacy and safety of vitamin K antagonists (VKAs) among atrial fibrillation (AF) patients are enhanced when the International Normalised Ratio (INR) is 2.0–3.0. Anticoagulation control among older patients is perceived to be lower and contributes to poorer initiation and uptake.

**Objective:**

To examine the quality of INR control, adverse clinical outcomes, and factors associated with bleeding in older AF patients (≥80 years).

**Methods:**

Anticoagulation control assessed by time in therapeutic range (TTR) (Rosendaal method) and percentage INRs in range (PINRR). Among the 205 patients aged ≥80 years, 58.5% were female, with mean (SD) CHA_2_DS_2_-VASc 4.4 (1.3) and HAS-BLED 1.8 (0.8) scores.

**Results:**

Mean (SD) TTR and PINRR were similar for those aged ≥80 vs. <80 years (66.7 (13.8) vs. 66.7 (13.1)) despite significantly lower INR monitoring intensity (51.2 (22.7) vs. 60.7 (25.8)) and shorter follow-up (4.4 (2.6–6.2) vs. 5.7 years (3.3–7.1)) in those ≥80 years of age. Good anticoagulation control (TTR and PINRR ≥70%) of 44% was seen in both age groups. No significant differences in composite major adverse clinical events were evident for those aged ≥80 vs. <80 years (*p* = 0.55). Cox regression analysis confirmed that age ≥80 years was associated with higher risk of bleeding (HR 1.90 (1.01–3.56); *p* = 0.047).

**Conclusions:**

Suboptimal (TTR and PINRR <70%) anticoagulation control was evident in all patients. Risk of bleeding increased, but there was no difference in thromboembolic events and all-cause mortality in those aged ≥80 years. Improving TTR to ≥70% and enhancing anticoagulation monitoring of VKA use remain a clinical priority to prevent bleeding complications, particularly among those aged 80 years and above.

## 1. Introduction

The prevalence of atrial fibrillation (AF) escalates as age increases, with rates of 0.12%–0.16% in those aged ≤49 years rising to 3.7%–4.2% and 10%–17% among those aged 60–70 years and >80 years, respectively [1]. Oral anticoagulation (OAC) is recommended for AF patients with a CHA_2_DS_2_-VASc score of 2 or more, and therefore, all AF patients aged 75 years and above should receive OAC for stroke prevention unless there is an absolute contraindication [2, 3]. However, anticoagulation in older people with AF is often challenging due to the physician's fear of bleeding complications resulting in undertreatment in up to 70% of patients [4–6].

The increased risk of bleeding with OAC therapy in older patients is attributable to several reasons: they have more comorbid conditions, thus requiring more medications, which in turn places them at greater risk of drug interactions [7]. Older people also have reduced metabolic clearance of drugs [7], and there is a risk of drug accumulation, thus predisposing them to side effects such as bleeding. Lastly, older people have higher risk of cognitive dysfunction [8, 9] which could affect medication adherence [10, 11] due to forgetfulness or by taking incorrect or higher than the prescribed doses leading to increased risk of bleeding tendency. Also, VKA therapy may be more difficult to manage among older people due to the frequency of INR monitoring required (which may be more problematic if it requires travel to an anticoagulant clinic), dietary intake, and drug interactions [12].

Among the studies with older (≥75 years) anticoagulated patients with AF, the randomised controlled trial of Warfarin versus Aspirin for Stroke Prevention in Octogenarians with atrial fibrillation (WASPO) [13] and the Birmingham Atrial Fibrillation Treatment of the Aged Study (BAFTA) [14] trials reported a mean percentage of INR within the range of 2.0–3.0 (PINRR) of 69.2 and 67 among their cohorts, respectively. However, two other analyses of older Italian AF patients aged ≥80 [15] and ≥75 years [16] reported better TTR (mean TTR 71% in both studies) in their cohorts of older AF patients. None of these studies [15–17] investigated the association of age with TTR and clinical outcomes.

Therefore, the aims of this study were, first, to examine the quality of vitamin K antagonist (VKA) control evidenced by time in the therapeutic range (TTR), second, to identify adverse clinical outcomes, and third, to identify factors associated with bleeding events in older (≥80 years) patients with AF.

## 2. Materials and Methods

This retrospective cohort analysis of older (≥80 years) patients with AF patients receiving VKA therapy for stroke prevention is a preplanned subgroup analysis from an earlier study [18] investigating anticoagulation control among different ethnic groups at one acute trust in the West Midlands, United Kingdom. A software programme utilised by the anticoagulant service to manage patients (DAWN AC®) was used to retrieve information on VKA therapy (target INR 2.0–3.0) received by AF patients for stroke prevention. AF patients were selected at random from the alphabetical list of patients (*n* = 2478) generated by this database. Of the 1070 patients selected, those with valvular heart disease (*n* = 45), unknown medical history (*n* = 13), and ethnicity not recorded (*n* = 21) were excluded, resulting in 991 participants included in the current analyses. The cohort was divided into those aged ≥80 years and those aged <80 years. The Strengthening the Reporting of Observational Studies in Epidemiology (STROBE) reporting guideline was followed to perform the data analysis and reporting of the study.

Electronic health records (CDA) were utilised to ascertain the demographic and clinical characteristics including comorbid diseases, laboratory results, medications, and the types of AF including paroxysmal, persistent, long-standing persistent, and permanent at baseline, with assumptions made based on the ECG recording available and length of time since AF diagnosis, according to the European Society of Cardiology (ESC) AF guidelines [2]. Smoking history was available for 717 (72.4%) patients. Chronic kidney disease was defined as present if patients had serum creatinine >200 umol/L or eGFR <60 ml/min; liver disease if alanine transaminase/alkaline phosphatase (ALT/ALP) >*x*3 upper limit of normal (ULN); and anaemia if haemoglobin level was <115 g/L for females and <135 g/L for males. The risk of stroke and bleeding and anticoagulant control was calculated from available information at baseline using CHA_2_DS_2_-VASc score [19], HAS-BLED score [20], and SAMe-TT_2_R_2_ score [21], respectively. The follow-up time was the entire period of VKA use until 31^st^ December 2016 or OAC therapy cessation.

### 2.1. Time in Therapeutic Range (TTR)

The electronic medical records and anticoagulation database were utilised to obtain all available INR results for the calculation of anticoagulation control (TTR and PINRR) from inception to 31^st^ December 2016 or OAC therapy cessation. The Rosendaal and PINRR methods were utilised to obtain the quality of INR control (INR 2.0–3.0), whereby the former calculates the proportion of time within the therapeutic range of INR (TTR) 2.0–3.0 using linear interpolation methods and the latter determines the proportion of INR within the therapeutic range by dividing the number of INRs in range with the total INR values [22, 23]. TTR was further stratified into TTR ≥70% and TTR <70%, according to a recent European consensus document for optimal efficacy and safety outcomes whilst on a VKA [24]. The percentage of subtherapeutic (INR <2.0) and supratherapeutic (INR >3.0) INRs, INRs >5.0, and INRs >8.0 was also determined.

### 2.2. Adverse Clinical Outcomes

In this study, adverse clinical outcomes were defined as the occurrence of stroke, transient ischemic attack (TIA), systemic embolism, the combination of major and clinically relevant nonmajor bleeding (CRNMB) as a “bleeding event,” CV hospitalisation, and all-cause death. These were collected from the electronic medical records. Stroke/TIA, systemic embolism, bleeding, cardiovascular hospitalisation, and death were then combined as composites of ≥1 major adverse clinical events (MACE). Definitions of each type of event can be found in an earlier publication [18]. This study received institutional review board approval and conformed to the Declaration of Helsinki.

### 2.3. Statistical Analysis

Baseline characteristics and adverse clinical outcomes are presented descriptively. Comparisons between the two age groups (≥80 vs. <80 years) were tested with the chi-square test. For continuous variables, the independent *t*-test was used for normally distributed data while the Mann–Whitney test was used for nonparametric data. Survival analysis is displayed using Kaplan–Meier curves, while Cox regression analysis (univariate and multivariate) was used to investigate factors associated with the risk of bleeding during follow-up (hazard ratios (HRs) and 95% confidence intervals (CIs)). *p* values <0.05 were considered statistically significant. All analyses were conducted using SPSS version 23.0 (IBM, NY, USA) [25].

## 3. Results

Baseline characteristics of the population grouped by ≥80 years and <80 years of age are shown in Table 1. There were 205 patients (20.6%) aged ≥80 years, and the majority were female (58.5%; *p* < 0.001) and of white ethnicity (85.9%; *p* = 0.016). Hypertension (85.9%; *p* = 0.011) and chronic kidney disease (50.2%; *p* = <0.001) were significantly more prevalent among patients aged ≥80 years, whereas smoking history (48.4%; *p* = 0.002) was significantly more prevalent in patients aged <80 years. As expected, the mean (SD) CHA_2_DS_2_-VASc score (4.4 (1.3); *p* < 0.001) and HAS-BLED score (1.8 (0.8); <0.001) were significantly higher among older patients, whereas the mean SAMe-TT_2_R_2_ score was significantly higher in the younger population.

**Figure 1:**
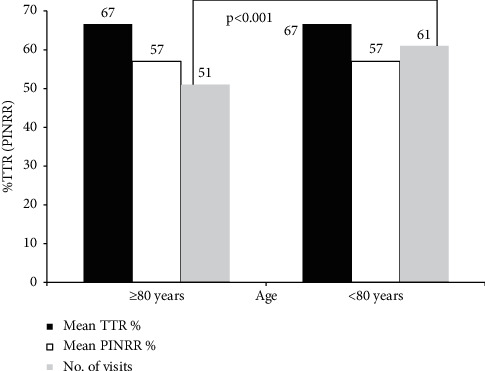
Mean percentage TTR and PINRR and number of visits among patients aged ≥80 and <80 years.

Most (96%) patients were VKA-naïve at baseline, and almost all (99.7%) were prescribed warfarin.  Table 2 presents the measures of anticoagulation control in the overall population and according to age ≥80 and <80 years. The quality of anticoagulation control by both measures, TTR (Rosendaal method) (66.6% in the ≥80 and <80 years age group) and PINRR (57.1% in the ≥80 years age group vs. 57.7% in the <80 years age group), was similar between the two age categories. Patients aged ≥80 years had significantly fewer INR visits (mean 51 vs. 61 visits; *p* < 0.001) compared to those aged <80 years and a lower duration of follow-up (Figure 1). Good TTR (defined as TTR and PINRR ≥70%) was 44% (TTR) and 14% (PINRR) in both age groups; over half of those aged ≥80 years did not achieve the optimal percentage TTR advocated by clinical guidelines (Table 2). No significant differences in subtherapeutic or supratherapeutic INRs were observed by age group (≥80 and <80 years).

Only 12 (6%) patients aged ≥80 years experienced thromboembolic events; 21 (10.2%) had a bleeding event; and eight (4.0%) died. Analogous figures for those aged <80 years were 38 (4.8%), 57 (7.3%), and 15 (1.9%), respectively. No significant differences in cardiovascular hospitalisations (23.9% vs. 18.5%) or the composite of major adverse clinical event (≥1 MACE) (33.7% vs. 31.2%) were evident between those aged ≥80 and <80 years, respectively (Table 3).

**Figure 2:**
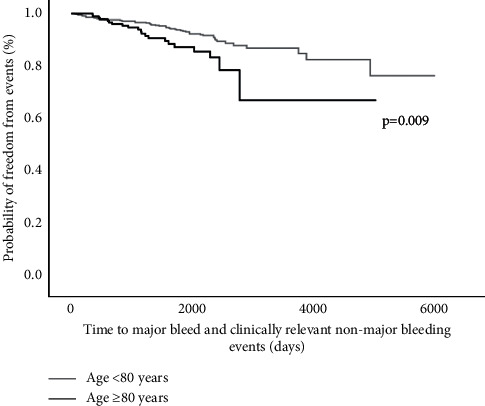
Kaplan–Meier curve of bleeding events among patients aged ≥80 and <80 years.

The Kaplan–Meier curve illustrates that the rate of bleeding events was significantly higher in those aged ≥80 years compared to those aged <80 years (2.4/100 pt-years vs. 1.3/100 pt-years, respectively) (log rank test: 6.73; *p* = 0.009 Figure 2). Univariate Cox regression analysis showed that only age ≥80 years (HR 1.93 (1.16–3.20); *p* = 0.01) was associated with bleeding risk, and this relationship persisted after adjusting for demographic and clinical variables (≥80 years: HR 1.90 (1.01–3.56); *p* = 0.047) (Supplementary Table 1).

## 4. Discussion

The main finding of this study is that anticoagulation control, evidenced by TTR and PINRR, was similar among those aged ≥80 and <80 years. Moreover, less than half (44%) of the older patients had optimal TTR (TTR ≥70%). Exploratory analyses showed that there were no significant differences in the composite endpoints between those aged ≥80 years and those aged <80 years, although there was a significantly higher bleeding risk even after adjustment for demographics and clinical variables among those aged ≥80 years.

These results are consistent with the data obtained from the BAFTA [13] and WASPO [14] trials (*n* = 973, aged >75 [13] and *n* = 75, aged >80 [14], respectively), with mean TTR comparable to the current older (≥80 years) cohort (mean TTR 67% in BAFTA [13] and 69% in WASPO [14], respectively, vs. 67% in the current older cohort). Two other Italian studies [15, 16] reported slightly higher mean TTR in their cohort (mean TTR 71% in both studies [15, 16] vs. 67% in the current older cohort). This could be due in part to the different study designs and cohorts of patients being investigated. Both studies [15, 16] utilised a prospective study design involving AF [15] and a variety of patients [16] (AF, venous thromboembolism (VTE), ischemic heart disease (IHD), valvular heart disease (VHD), and arterial vascular patients) from the anticoagulant clinic, compared to the current study which had a retrospective design involving AF patients only.

Conversely, mean TTR was lower in another study [17] of 472 AF patients managed by an on-site anticoagulation clinic among older people (mean age 77 (65–97) years; mean TTR 58%). This may be explained by the inclusion of an inception cohort (period of VKA treatment first year of therapy) [17], whereas the current study included patients throughout the entire period of treatment (median duration of VKA treatment 5.2 years reflecting long-term VKA management). Differences in the demographics and clinical characteristics might also partly explain the reasons of lower anticoagulation control in Hylek et al.'s study [17] compared to the current study (32% [17] vs. 20% of ≥80 years; 39% [17] vs. 4.9% concurrent antiplatelet use, respectively) reflecting greater risk factors for bleeding in Hylek et al.'s study [17] compared to the present study. Low TTR (mean TTR 48%) has also been reported in another inception cohort study [26] suggesting the difficulties in achieving good control with VKA therapy may be heightened, especially during the inception period [26]. Observations from the current study and other studies [13, 14, 17, 26] show suboptimal anticoagulation control among the very old, which could be due to the frequency of INR monitoring required (which may be more problematic if it requires travel to an anticoagulant clinic), inclusion of an inception cohort, presence of risk factors for bleeding, dietary intake, and drug interactions [12], all of which could influence the quality of anticoagulation control in this age group.

Exploratory analyses of adverse outcomes showed no significant differences in the composite endpoints (≥1 MACE) between those aged ≥80 years and those aged <80 years. However, those age ≥80 years had a significantly higher risk of bleeding after adjustment for demographic and clinical variables. Previous studies have reported conflicting results regarding the increased risk of bleeding among older patients on OAC therapy. In one Italian study, the absolute rate of major bleeding was 2.5 vs. 0.9 per patient years among AF patients aged ≥80 vs. <80 years, respectively, receiving warfarin therapy [15]. Conversely, a 5-fold increase in the incidence rate of bleeding was reported in those aged ≥80 years compared to <80 years (13.1 vs. 4.8 per 100 patient-years, respectively) in another study [17]. Age ≥80 years was associated with increased risk of bleeding events in both studies [15, 17]. The differences in bleeding rates between these studies might be explained by the higher proportion of patients with CAD (35% vs. 20%) who were prescribed concomitant aspirin therapy (40% [17] vs. 35% [15], respectively). Indeed, increased age and concomitant antiplatelet use are both factors known to increase risk of bleeding. More contemporary studies have compared the risk of major bleeding among AF patients on VKAs and NOAC. Patti et al. [27] reported on 3825 AF patients (aged ≥75 years) from two large, real-world prospective European registries comparing clinical outcomes on NOACs vs. VKA and demonstrated an increase in the rate of major bleeding among those on VKA compared to NOAC (3.8 vs. 2.7 per 100 patient years; adjusted OR 0.58; 95% CI 0.38–0.90; *p* = 0.013, respectively). In contrast, a meta-analysis (22 studies, *n* = 440,281) comparing the safety and efficacy of NOACs vs. VKA among AF patients aged ≥75 years found no differences in major bleeding (HR 0.94; 95% CI 0.85–1.05) [28].

Close attention needs to be paid to older patients receiving OAC therapy to mitigate bleeding complications. Various bleeding scores are available to assess bleeding risk in AF patients [29]. These scores can be used to guide physicians to identify factors that may predispose patients to bleeding [30]. Any modifiable risk factors for bleeding, such as uncontrolled hypertension, labile INR, alcohol excess, and nonessential concomitant antiplatelet therapy, should be addressed. The risk of bleeding is not static [31] and, therefore, needs to be evaluated periodically [2, 32].

### 4.1. Strengths and Limitations

To our knowledge, this is the first large, nonrandomised controlled trial cohort study in the United Kingdom to assess anticoagulation control and explore adverse clinical outcomes in the very old. Hence, it may be more representative of “real-world” AF patients. Most studies examining differences in anticoagulation control between older and younger patients were conducted in the United States and European countries [13–16] and included an inception cohort [17] which might impact the quality of anticoagulation control with VKA therapy. Furthermore, TTR was calculated using many INR results (mean (SD) 58.7 (25.5)) for a median of 5.2 (3.2–7.0) years of follow-up reflecting the long-term quality of anticoagulation control in this centre.

However, the retrospective review from medical records means that some information (patients' ethnic group, medical history, and medication history) was not readily available for a small proportion (3.2%) and they had to be excluded. In addition, only adverse clinical events captured in the hospital electronic health records were captured which might have led to underestimation of the actual number of adverse events.

## 5. Conclusions

Suboptimal (TTR and PINRR <70%) anticoagulation control was evident in all patients. There was increased risk of bleeding but no difference in thromboembolic events and all-cause mortality in those aged ≥80 years. Therefore, optimising anticoagulation control by improving TTR to ≥70% and enhancing anticoagulation monitoring of VKA use remains a clinical priority to prevent bleeding complications, particularly among those aged 80 years and above.

## Figures and Tables

**Table 1:** Baseline characteristics of the study population overall and by age group (≥80 vs. <80 years).

	Total, *N* = 991	Age ≥80 years, *N* = 205	Age <80 years, *N* = 786	*p* value
Mean (SD) age	71.6 (9.4)	—	—	—
Female	443 (44.7)	120 (58.5)	323 (41.1)	<0.001
Male	548 (55.3)	85 (41.5)	463 (58.9)	
*Ethnicity*				
White	807 (81.4)	176 (85.9)	631 (80.3)	0.016
South-Asian	102 (10.3)	10 (4.9)	92 (11.7)	
Afro-Caribbean	82 (8.3)	19 (9.3)	63 (8.0)	
*Medical history*				
Heart failure	138 (13.9)	31 (15.1)	107 (13.6)	0.66
Hypertension	785 (79.2)	176 (85.9)	609 (77.5)	0.011
Diabetes mellitus	204 (20.6)	38 (18.5)	166 (21.1)	0.47
Stroke/TIA	179 (18.1)	40 (19.5)	139 (17.7)	0.61
VTE	38 (3.8)	7 (3.4)	31 (3.9)	0.88
PAD	26 (2.6)	8 (3.9)	18 (2.3)	0.30
Vascular disease^†^	163 (16.4)	37 (18.0)	126 (16.0)	0.56
Lung disease^‡^	196 (19.8)	34 (16.6)	162 (20.6)	0.23
Cardiomyopathy^§^	30 (3.0)	4 (2.0)	26 (3.3)	0.77
Chronic kidney disease^††^	370 (37.3)	103 (50.2)	267 (34.0)	0.12
Anaemia	145 (14.6)	34 (16.6)	111 (14.1)	<0.001
Smoker/ex-smoker (*N* = 717)	326 (45.5)	49 (33.8)	277 (48.4)	<0.001
*Type of AF*				
Paroxysmal	274 (27.6)	48 (23.4)	226 (28.8)	0.004
Persistent	229 (23.1)	47 (22.9)	182 (23.2)	
Permanent	488 (49.2)	110 (53.7)	378 (48.1)	
*Medication*				
ACEI/ARB	561 (56.6)	115 (56.1)	446 (56.7)	0.43
Beta-blocker	455 (45.9)	87 (42.4)	368 (46.8)	0.10
CCB	350 (35.3)	82 (40.0)	268 (34.1)	<0.001
Digoxin	226 (22.8)	43 (21.0)	183 (23.3)	0.15
Diuretics	439 (44.3)	120 (58.8)	319 (40.6)	0.07
Amiodarone	58 (5.9)	7 (3.4)	51 (6.5)	0.25
Concurrent antiplatelet	46 (4.6)	10 (4.9)	36 (4.6)	0.53
Mean (SD) CHA_2_DS_2_-VASc score	3.4 (1.6)	4.4 (1.3)	3.1 (1.6)	<0.001
Mean (SD) HAS-BLED score	1.5 (0.9)	1.8 (0.8)	1.5 (0.9)	<0.001
Mean SAMe-TT_2_R_2_ score	2.3 (1.4)	2.2 (1.2)	2.4 (1.4)	0.04

ACEI/ARB: angiotensin-converting enzyme inhibitor/angiotensin receptor blockade; CCB: calcium channel blocker; CHA_2_DS_2_-VASc score**:** Congestive heart failure/left ventricular dysfunction, Hypertension, Age ≥75 years (2 points), Diabetes, Stroke (2 points), Vascular disease, Age 65–74 years, and Sex category (female). Total scores range between 0–9; low-risk CHA_2_DS_2_-VASc score: 0, intermediate: 1, high-risk CHA_2_DS_2_-VASc score: ≥2; TIA: transient ischemic attack; TE: thromboembolism; HAS-BLED score: uncontrolled Hypertension: systolic ≥160 mmHg, Abnormal renal/liver function, Stroke, Bleeding history or predisposition, Labile INR ratio/TTR <60, Drugs/alcohol concomitantly. Total scores range between 0–9; low risk of bleeding ranges between 0–2 and high risk of bleeding ≥3; SAMe-TT_2_R_2_ score: Sex female, Age<60, Medical history (more than two comorbidities), Treatment (interacting drug, e.g., Amiodarone), Tobacco use (doubled), and Race (nonwhite, doubled). Total scores ranged from 0–8; probable good response to VKA therapy range between 0–2; and probable poor response to VKA therapy ranged from ≥3. †Vascular disease: prior myocardial infarction, peripheral artery disease, or aortic plaque; ‡lung disease includes obstructive and restrictive diagnosed lung conditions; §cardiomyopathy: dilated, restrictive, and obstructive myocardial conditions; ††kidney disease: eGFR<60 ml/min.

**Table 2:** Measures of anticoagulation control among the overall population and in patients aged ≥80 and < 80 years.

*N* (%)	Total, *N* = 991	Age ≥80, *N* = 205	Age <80, *N* = 786	*p* value
Mean TTR (SD)	66.6 (13.2)	66.6 (13.8)	66.6 (13.1)	1.00
TTR <70%	550 (55.5)	114 (55.6)	436 (55.5)	1.00
TTR ≥70%	441 (44.5)	91 (44.4)	350 (44.5)	
Mean PINRR (SD)	57.6 (11.2)	57.1 (11.6)	57.7 (11.1)	0.54
PINRR <70%	851 (85.9)	176 (85.9)	675 (85.9)	1.00
PINRR ≥70%	140 (14.1)	29 (14.1)	111 (14.1)	
Mean (SD) number of visits	58.7 (25.5)	51.2 (22.7)	60.7 (25.8)	<0.001
Mean (SD) percentage of INRs <2	25.7 (10.0)	26.6 (9.8)	25.5 (24.5)	0.17
Mean (SD) percentage of INRs >3	16.6 (7.2)	16.4 (15.6)	16.7 (7.1)	0.60
INR >5	293 (29.6)	70 (34.1)	223 (28.4)	0.13
INR >8	41 (4.1)	10 (4.9)	31 (3.9)	0.69
Median (IQR) years of follow-up	5.2 (3.2–7.0)	4.4 (2.6–6.2)	5.7 (3.3–7.1)	<0.001

INR: International Normalised Ratio; IQR: interquartile range; PINRR: percentage of INRs within range; SD: standard deviation; TTR: time in therapeutic range.

**Table 3:** Major adverse clinical outcomes among patients receiving warfarin for stroke prevention in AF, overall and in patients aged ≥80 and < 80 years.

Outcomes, *N* (%)	Age ≥80, *N* = 205	Event rate/100 pt-yrs	Age <80, *N* = 786	Event rate/100 pt-yrs	*p* value for proportions
≥1 MACE	64 (31.2)	8.4	265 (33.7)	7.4	0.55
Stroke/TIA/SE	12 (5.9)	1.4	38 (4.8)	0.9	0.68
Bleeding^*∗*^	21 (10.2)	2.4	57 (7.3)	1.3	0.16
Cardiovascular hospitalisation^‡^	38 (18.5)	4.7	188 (23.9)	5.0	0.12
Death	8 (3.9)	0.9	15 (1.9)	0.3	0.15

MACE: major adverse clinical events, SE: systemic embolism, TIA: transient ischemic attack, yrs: years. ^*∗*^Bleeding is combination of major bleed according to the International Society on Thrombosis and Haemostasis (ISTH) and clinically relevant nonmajor bleed (CRNMB). ‡Cardiovascular hospitalisation: a hospitalisation with a cardiovascular cause: (i) heart failure, myocardial infarction, new angina, nonfatal cardiac arrest, ventricular arrhythmia, uncontrolled atrial fibrillation/atrial flutter, and supraventricular arrhythmia; (ii) valve surgery, coronary artery bypass graft surgery (CABG), percutaneous transluminal coronary angioplasty (PTCA) surgery, pacemaker/ICD insertion, carotid endarterectomy, peripheral angioplasty/surgery, and limb amputation and as recorded in the patient's medical documents; DVT: deep vein thrombosis; major bleeding: ISTH major bleeding: fatal bleeding and/or symptomatic bleeding in a critical area or organ, such as intracranial, intraspinal, intraocular, retroperitoneal, intraarticular or pericardial or intramuscular with compartment syndrome and/or bleeding causing a fall in the haemoglobin level of 2 g/dL (1.24 mmol/L) or more or leading to transfusion of two or more units of whole blood or red cells; clinically relevant nonmajor bleeding (CRNMB): clinically overt bleeding not satisfying the criteria for major bleeding and that led to hospitalisation, physician medical or surgical treatment, or a change in antithrombotic therapy; PE: pulmonary embolism; SE: systemic embolism; TIA: transient ischemic attack; VTE: venous thromboemboli.

## Data Availability

Data sharing is restricted due to institutional policies.

## Additional Points

*What Is Already Known*. Anticoagulation control among older people perceived to be poorer and results in lower anticoagulation initiation and uptake.

*What This Paper Adds*. (i) Similar TTR and major adverse clinical events in those aged ≥80 years but higher bleeding risk. (ii) Intensity of anticoagulation monitoring significantly lower among those aged ≥80 years.
