# Supplementary material for: Anticoagulation Control in Older Atrial Fibrillation Patients Receiving Vitamin K Antagonist Therapy for Stroke Prevention
Source: Int J Clin Pract. 2022 Jan 31;2022:5951262. doi: 10.1155/2022/5951262 (PMC9159113; doi:10.1155/2022/5951262)
Supplement: Supplementary Materials — Supplementary Table 1. [file 5951262.f1.docx]

**Supplementary Table 1:** Cox proportional hazard regression analysis for all bleeding events, including major bleeding and clinically relevant non-major bleeding including age ≥80 years

|  | **Univariate** | | **Multivariate** | |
| --- | --- | --- | --- | --- |
|  | **HR (95% CI)** | **p-value** | **HR (95% CI)** | **p-value** |
| Age ≥80 years | **1.93 (1.16-3.20)** | **0.01** | **1.90 (1.01-3.56)** | **0.047** |
| Female sex | 0.92 (0.58-1.45) | 0.71 | 0.82 (0.47-1.44) | 0.49 |
| Smoking history | 0.86 (0.51-1.45) | 0.57 | 0.91 (0.51-1.61) | 0.75 |
| Ethnicity† | 1.13 (0.64-2.02) | 0.67 | 1.01 (0.49-2.05 | 0.98 |
| Hypertension | 1.31 (0.73-2.33) | 0.37 | 1.54 (0.72-3.31) | 0.27 |
| Stroke/TIA history | 1.10 (0.63-1.93) | 0.74 | 1.09 (0.56-2.13) | 0.80 |
| Heart failure | 1.38 (0.78-2.47) | 0.27 | 1.71 (0.91-3.24) | 0.10 |
| Diabetes mellitus | 1.18 (0.69-2.02) | 0.54 | 0.93 (0.47-1.84) | 0.84 |
| Vascular disease | 0.97 (0.51-1.83) | 0.92 | 0.78 (0.35-1.77) | 0.56 |
| Chronic kidney disease | 0.92 (0.57-1.47) | 0.73 | 0.65 (0.36-1.17) | 0.15 |
| Anaemia | 1.46 (0.80-2.65) | 0.22 | 1.67 (0.78-3.61) | 0.19 |
| Bleeding history | 1.48 (0.68-3.23) | 0.32 | 0.99 (0.37-2.64) | 0.99 |
| Concomitant antiplatelet therapy | 1.47 (0.54-4.03) | 0.45 | 0.92 (0.21-44.06) | 0.91 |
| TTR <70% | 1.52 (0.95-2.42) | 0.08 | 1.74 (0.99-3.05) | 0.055 |

CI: confidence interval; HR: hazard ratio; TIA: transient ischemic attack; TTR: time in therapeutic range; † comparison was White vs. other ethnic groups
